# Targeting caspase-8: a new strategy for combating hepatocellular carcinoma

**DOI:** 10.3389/fimmu.2024.1501659

**Published:** 2024-12-12

**Authors:** Haoran Chen, Yumeng Lin, Jie Chen, Xuemei Luo, Yubo Kan, Yuqi He, Renhe Zhu, Jiahui Jin, Dongxuan Li, Yi Wang, Zhongyu Han

**Affiliations:** ^1^ Department of General Surgery, Chengdu Xinhua Hospital Affiliated to North Sichuan Medical College, Chengdu, China; ^2^ Health Management Center, Nanjing Tongren Hospital, School of Medicine, Southeast University, Nanjing, China; ^3^ Sichuan Provincial Woman’s and Children’s Hospital/The Affiliated Women’s and Children’s Hospital of Chengdu Medical College, Chengdu, China; ^4^ Department of Blood Transfusion, Lu’an People’s Hospital, the Affiliated Hospital of Anhui Medical University, Lu’an, China; ^5^ Department of gastroenterology, Baoji Central Hospital, Baoji, China

**Keywords:** caspase-8, hepatocellular carcinoma, apoptosis, necroptosis, pyroptosis, PANoptosis, tumor microenvironment

## Abstract

Hepatocellular carcinoma (HCC) represents the most prevalent form of primary liver cancer and has a high mortality rate. Caspase-8 plays a pivotal role in an array of cellular signaling pathways and is essential for the governance of programmed cell death mechanisms, inflammatory responses, and the dynamics of the tumor microenvironment. Dysregulation of caspase-8 is intricately linked to the complex biological underpinnings of HCC. In this manuscript, we provide a comprehensive review of the regulatory roles of caspase-8 in apoptosis, necroptosis, pyroptosis, and PANoptosis, as well as its impact on inflammatory reactions and the intricate interplay with critical immune cells within the tumor microenvironment, such as tumor-associated macrophages, T cells, natural killer cells, and dendritic cells. Furthermore, we emphasize how caspase-8 plays pivotal roles in the development, progression, and drug resistance observed in HCC, and explore the potential of targeting caspase-8 as a promising strategy for HCC treatment.

## 1 Introduction

Cancer has been a grave health issue worldwide for a long time. Through remarkable advancements in medical technology, the overall survival rates for numerous cancers have significantly improved in comparison with those reported in previous decades. Nevertheless, the survival rates for certain cancers, such as liver cancer, still fall short of satisfactory levels. Liver cancer ranks among the deadliest of malignancies, with a 5-year relative survival rate of only 22% (1). Hepatocellular carcinoma (HCC), in particular, is the foremost subtype of liver cancer, accounting for approximately 90% of all primary liver cancer cases (2). The primary risk factors contributing to HCC differ across geographical locations; however, they commonly include viral hepatitis types B and C (HBV, HCV), alcohol-related liver diseases, nonalcoholic fatty liver diseases, autoimmune hepatitis, and other related conditions (3). Regardless of the specific aetiology, ongoing damage to hepatocytes is a central factor in the development of chronic hepatitis, liver fibrosis, cirrhosis, and ultimately, the development of HCC (4). To maintain the normal functionality and homeostasis of the liver, hepatocytes require ongoing renewal and repair, and damaged hepatocytes are eliminated through programmed cell death (PCD), which helps to prevent the accumulation of potentially harmful mutations (5). However, the persistent PCD of hepatocytes results in the emission of damage-associated molecular patterns (DAMPs). These molecular signals, in turn, go on to trigger immune cell activation and inflammatory responses. This establishes a vicious inflammation-PCD cycle that exacerbates liver injury (5). Moreover, triggering PCD in tumor cells is a vital component of radiotherapy and chemotherapy for treating HCC (6). The unfavorable prognosis of HCC is closely associated with the persistent presence of cirrhosis and resistance to radiotherapy/chemotherapy. Therefore, targeting the crucial links in PCD can significantly increase the therapeutic effect on HCC, decrease the recurrence rate, and ultimately lower the mortality rate.

Caspase-8, a cysteinyl aspartate specific proteinase (caspase), plays a central role in a myriad of signaling pathways and is crucial for the regulation of PCD, immune cell homeostasis, and cytokine production (7). In HCC, dysregulation of caspase-8 expression is often observed, leading to functional imbalances within HCC cells and the tumor microenvironment (TME) (8, 9). This imbalance can have profound consequences for the progression, aggressiveness, and drug resistance of HCC (8). Consequently, a comprehensive understanding of the roles and regulatory mechanisms of caspase-8 in the context of HCC is essential for crafting impactful treatment strategies aimed at combating this cancer.

This manuscript addresses in on the latest research developments, aiming to dissect how caspase-8 orchestrates the regulation of PCD, inflammation, and the TME. Furthermore, we explore the implications of caspase-8 in the aetiology of HCC and evaluates the potential of caspase-8 as a therapeutic strategy for HCC treatment.

## 2 Caspase family and caspase-8

The caspase family is classified within the interleukin-1β-converting enzyme family of proteases, which are crucial components of cellular processes (10, 11). Structurally, all members of the caspase family feature an active site containing a cysteine (12). During the process of peptide bond hydrolysis, these enzymes utilize the cysteine side chain as a nucleophile, allowing them to specifically cleave the peptide bond after the specific aspartic acid residue within the target protein (13, 14). This cleavage typically results in the activation or inactivation of the substrate rather than its complete degradation (15). In cells, caspases typically exist in an inactive zymogen form known as procaspases (16). Under specific conditions, procaspases undergoes dimerization or oligomerization, leading to their activation and the formation of caspases, which perform proteolytic functions (17). The proteolytic activity of caspases is achieved through their caspase domain (16). During the activation process, the protease effector domain of pro-caspase undergoes cleavage, yielding a large subunit (approximately 20 kDa) and a small subunit (approximately 10 kDa), subsequently forming an enzymatically active complex (18). The caspase family consists of 14 members (caspase-1 to -14). On the basis of their amino acid sequence homology and functions, caspases-1 to -13 are classified into apoptosis activators, apoptosis executioners and inflammatory subfamilies (6). Caspase-14 is unrelated to apoptosis and inflammation and is instead associated with epithelial cell differentiation (19).

Caspase-8, also known as FLICE, MACH, or Mch5, belongs to the caspase family (20, 21). Inside cellular structures, the default state of caspase-8 is that of its dormant precursor, procaspase-8 (22). This includes a C-terminal domain that consists of two subunits: a larger one, p18, and a smaller one, p10 (23). P18 houses an active catalytic cysteine residue, which is crucial for its enzymatic activity, and p10 acts as a substrate-binding domain that is responsible for recognizing and binding to specific target proteins (24). Additionally, at the N-terminus, procaspase-8 possesses two death effector domains (DED1 and DED2), which are instrumental in the initial recognition of upstream signals and the subsequent activation of the zymogen (25). During the activation process, procaspase-8 is recruited by an array of upstream signals, such as the death-inducing signaling complex (DISC), leading to the formation of dimers (26, 27). These dimers then undergo two rounds of self-cleavage, culminating in the assembly of an enzymatically active tetramer consisting of two p18 and two p10 subunits, which constitute the active form of caspase-8 (24).

Caspase-8 is a multifunctional protein that is instrumental in the complex control mechanisms of PCD, inflammation, and innate immune responses. In the subsequent sections, we further explore these pivotal functions and their potential implications.

## 3 Caspase-8 and PCD

### 3.1 Caspase-8 and apoptosis

Apoptosis is a pivotal form of PCD first characterized in 1972 (28). Morphologically, apoptotic cells undergo shrinkage, display nuclear disintegration, exhibit plasma membrane blebbing, and ultimately form distinct apoptotic bodies (28–30). Apoptotic cells do not release inflammatory mediators but are quickly phagocytosed by nearby macrophages, making apoptosis a low-immunogenic form of PCD (28). Caspases are the central component of apoptosis. Apoptosis initiates upon the reception of apoptotic signals, and depending on the different sources and triggering mechanisms of these signals, apoptosis is primarily divided into extrinsic and intrinsic pathways (31, 32). The intrinsic pathway (mitochondrial pathway) is activated in response to cellular stressors or injuries such as DNA damage and oxidative stress. Caspase-9 serves as the primary apoptosis activator in this pathway (31, 33). Conversely, the extrinsic pathway, commonly designated the death receptor (DR) pathway, is set into motion by extracellular apoptotic signals, with caspase-8 acting as the main apoptosis activator in this cascade (32). This pathway is initiated by the interaction of DRs (such as Fas and TNFR1), which are located on the cell surface, with their specific ligands (34). For example, upon binding with Fas ligand (FasL), Fas undergoes conformational alterations, facilitating the recruitment of the adaptor protein Fas-associated death domain (FADD) (35). FADD contains a DED and facilitates the assembly of procaspase-8 through the DED: DED interaction (36). As previously mentioned, procaspase-8 comprises DED1 and DED2. The interaction between FADD-DED and DED1 leads to the binding of pro-caspase-8 with FADD, culminating in the assembly of the DISC (37, 38). DED2 subsequently recruits an additional procaspase-8 and binds to its DED1, initiating dimerization and autocatalytic cleavage of procaspase-8 (39). The activated caspase-8 then cleaves multiple downstream target proteins, such as the apoptosis executioner caspases, thereby leading to activation of the extrinsic apoptosis cascade.

Moreover, caspase-8 is also instrumental in initiating the intrinsic apoptotic pathway. It achieves this by cleaving the Bcl-2 homology 3-interacting domain death agonist (BID) to generate a truncated BID (tBID), which subsequently binds to the Bcl-2-associated X protein (Bax) (40). This interaction precipitates alterations in mitochondrial membrane permeability and the release of cytochrome-c (cyt-c), culminating in the activation of caspase-9 and the induction of intrinsic apoptosis (41).

### 3.2 Caspase-8 and necroptosis

Necroptosis is a mixed-lineage kinase-like (MLKL)-dependent type of PCD. Once activated, MLKL enhances plasma membrane permeability, resulting in cell rupture, the liberation of intracellular contents, and the ensuing inflammatory reactions within necroptotic cells (42). Necroptosis is induced by upstream signals such as tumor necrosis factor receptor (TNFR) superfamily receptors, toll-like receptor (TLR)-3/4, and Z-DNA binding protein-1 (ZBP1) (43–46). Necroptosis is a type of caspase-8-independent PCD, yet caspase-8 plays a pivotal regulatory role in the process of necroptosis.

In the classical DR pathway, caspase-8 plays a role in inhibiting necroptosis. Using TNFR1 as an example, upon recognition of TNF-α, TNFR1 recruits receptor-interacting serine/threonine-protein kinase 1 (RIPK1) and the TNF receptor-associated death domain (TRADD) at its tail to form complex I (47, 48). In addition, proteins such as cellular inhibitor of apoptosis protein 1/2 (cIAP1/2), TNF receptor-associated factor 2/5 (TRAF2/5), transforming growth factor-β-activated kinase 1 (TAK1), and IκB kinases (IKKs) are also recruited and can regulate the activity of RIPK1 by modulating its posttranslational modifications, including deubiquitination, ubiquitination, and phosphorylation (49–52). Deubiquitination of RIPK1 promotes the dissociation of RIPK1 and TRADD from complex I and the formation of complex II (34).

**Figure 1:**
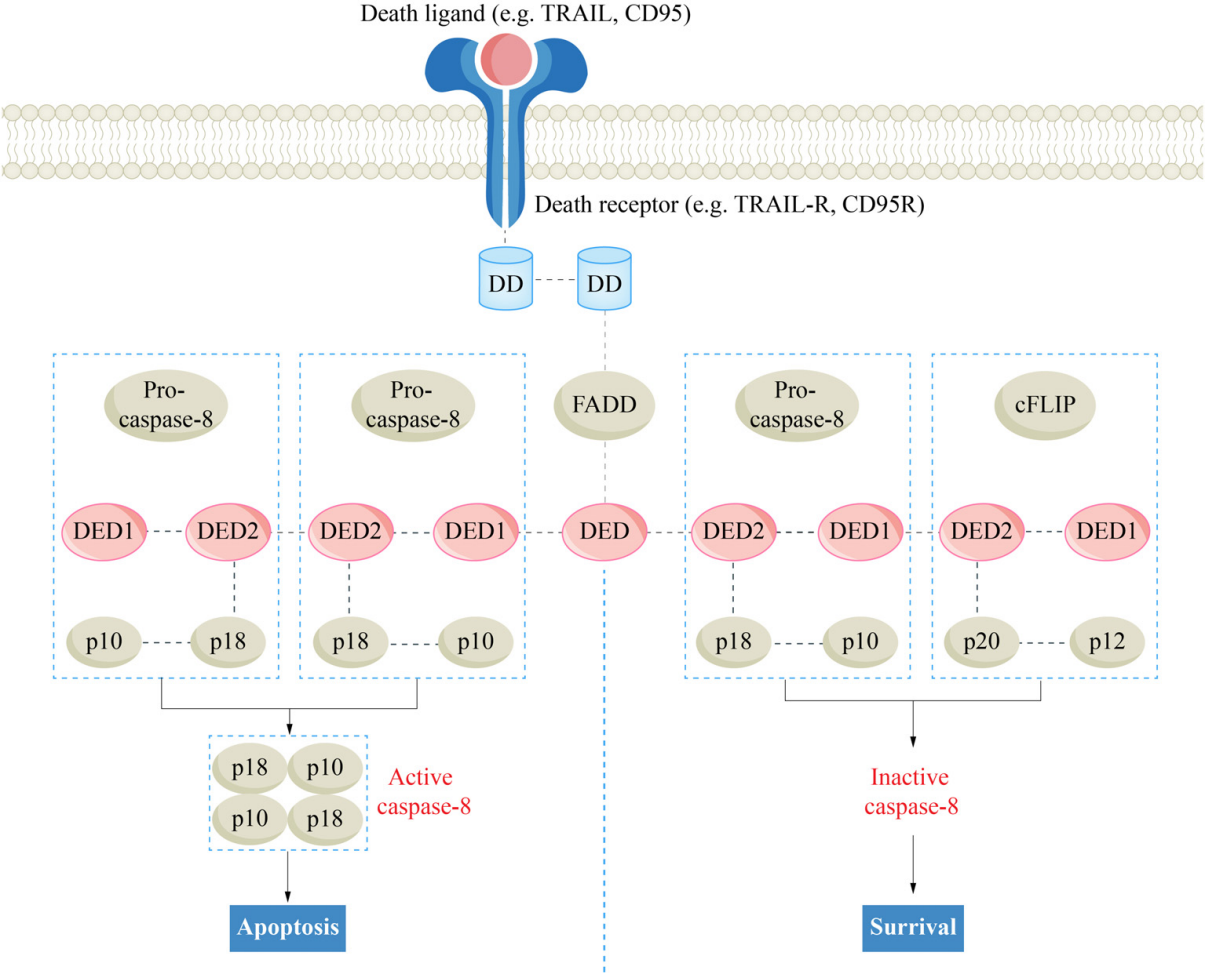
The regulatory effect of cFlip on death receptor pathway apoptosis. Caspase-8, cysteinyl aspartate specific proteinase 8; cFLIP, cellular-FLICE inhibitory protein; DD, death domain; DED, death effector domain; FADD, Fas-associated death domain.

Upon activation, caspase-8 initiates the assembly of the RIPK1-TRADD-FADD-caspase-8 complex (complex IIa), leading to apoptosis (53). Under conditions of high intracellular RIPK3 levels, the formation of complex IIb or IIc is dependent on the activity of caspase-8. When caspase-8 is activated, RIPK1-RIPK3-FADD-caspase-8 forms the rippoptosome (complex IIb) (54). In the rippoptosome, caspase-8 forms a heterodimer with cellular-FLICE inhibitory protein (cFLIP) to exert an inhibitory effect. cFLIP is a homologue of caspase-8 that lacks proteolytic activity (55). Owing to its low activity, the caspase-8-cFLIP heterodimer is capable of cleaving RIPK1, effectively blocking necroptosis (**Figure 1**) (56, 57). Additionally, this heterodimer suppresses the apoptotic-promoting function of caspase-8 while promoting cell survival (58, 59). In the absence of caspase-8, RIPK1-RIPK3-MLKL forms the necrosome (complex IIc), sequentially activating RIPK3 and MLKL, leading to the phosphorylation and oligomerization of MLKL, ultimately resulting in membrane disruption and necroptosis (60–62).

### 3.3 Caspase-8 and pyroptosis

Pyroptosis is a form of gasdermin (GSDM)-mediated PCD that plays a crucial role in innate immune responses and the elimination of pathogens (63, 64). The GSDM family comprises six members: GSDMA-E and PJVK. Among these, GSDMA-E feature two distinct structural domains, the N-terminal pore-forming domain (N-PFD) and the C-terminal regulatory domain (C-RD), which contribute to their unique functional roles (65, 66). The activated N-PFD mediates the formation of pores in the cell membrane, whereas the C-RD interacts with the N-PFD through a linker region to exert self-inhibition under physiological conditions (66). When the linker region is cleaved by upstream signals such as caspase and granzyme B, N-PFD is released, allowing GSDM to oligomerize at the plasma membrane and form pores, which facilitate the release of cellular contents and inflammatory mediators, ultimately triggering pyroptosis (66–68). Consequently, pyroptotic cells also display necrotic-like characteristics, including cell swelling and rupture (69).

Caspase-8 participates in the regulation of both the canonical and noncanonical pathways of pyroptosis. In the canonical pathway, an inflammasome is assembled through the interaction of pattern recognition receptors (PRRs), such as NOD-like receptor pyrin domain-containing protein 3 (NLRP3) and NLRC4, the adaptor protein ASC, and procaspase-1 (70). This complex activates caspase-1, leading to the cleavage of GSDMD and ultimately inducing pyroptosis. Furthermore, caspase-1 also mediates the cleavage of pro-IL-1β and pro-IL-18, thereby enhancing the activation and release of the inflammatory mediators interleukin-1 (IL-1β) and IL-18 (71). Caspase-8 can promote the classical pyroptosis pathway without relying on enzymatic activity. Research has shown that mutant caspase-8 (CASP8^C362A^), which lacks enzymatic activity, can promote ASC activation and activate caspase-1 (7). Phylogenetic analysis revealed that the DED2 domain of procaspase-8 and the pyrin domain (PYD) of ASC are located on the same branch (72). In the noncanonical pathway, caspase-8 can cleave GSDMC, GSDMD and GSDME to trigger pyroptosis. Caspase-8 can cleave GSDMD independently of caspase-1 and confer susceptibility to TNF-induced lethality (73). The metabolite α-KG elevates intracellular ROS levels, oxidizing DR6. Activated DR6 recruits caspase-8 and GSDMC, triggering the caspase-8-GSDMC pathway (74). In addition, in the presence of GSDMC and nuclear programmed cell death protein 1 (nPD-L1), TNF-α-activated caspase-8 can trigger pyroptosis through the caspase-8-GSDMC pathway (75). Furthermore, elevated TNF-α can also activate caspase-8 and caspase-3 through the DR pathway, triggering the transition from caspase-3-GSDME-mediated apoptosis to pyroptosis (76).

### 3.4 Caspase-8 and PANoptosis

**Figure 2:**
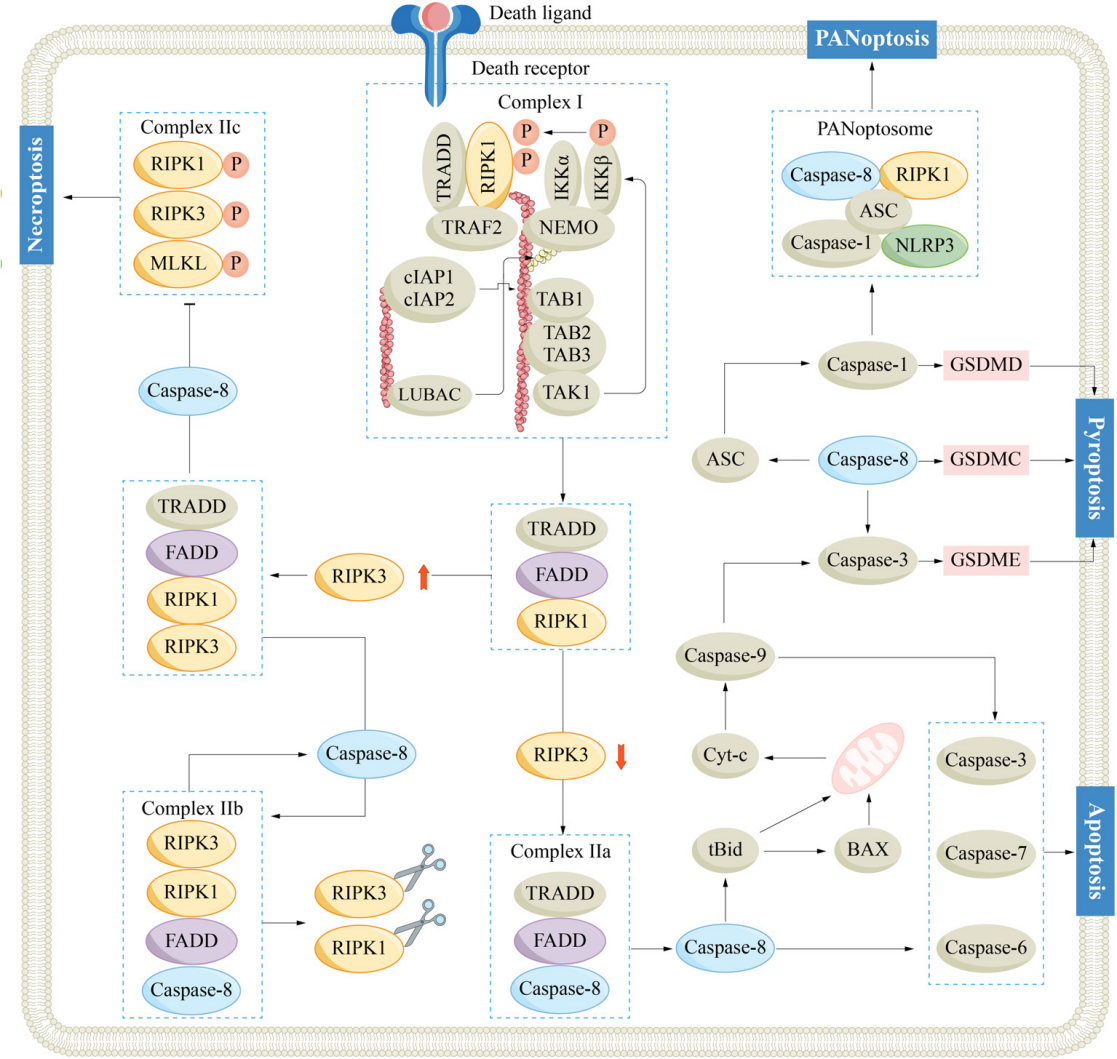
Mechanisms of caspase-8 regulated apoptosis, necroptosis, pyroptosis and PANoptosis. ASC, adaptor apoptosis-associated speck-like protein containing a CARD; BAX, Bcl-2-associated X protein; Bid, Bcl-2 homology 3 interacting domain death agonist; Caspase-1/3/6/7/8/9, cysteinyl aspartate specific proteinase 1/3/8/9; cIAP, cellular inhibitor of apoptosis protein; cyt-c, cytochrome-c; FADD, Fas-associated death domain; GSDM, gasdermin; IKK, IκB kinase; IL-1/18, interleukin-1/18; MLKL, mixed-lineage kinase-like; NEMO, nuclear factor-kappaB essential modulator; NLRP3, NOD-like receptor pyrin domain-containing protein 3; RIPK1/3, receptor-interacting serine/threonine-protein kinase 1/3; TAK, transforming growth factor-β-activated kinase 1; TNF, tumor necrosis factor receptor; TRADD, TNF receptor-associated death domain; TRAF2, TNF receptor-associated factor 2.

PANoptosis is a newly discovered type of PCD that possesses the key features of pyroptosis, apoptosis, and/or necroptosis, but its mechanism cannot be solely explained by these types of PCD (77). The PANoptosome serves as the molecular platform that triggers PANoptosis, and its assembly and activation are crucial for the simultaneous involvement of pyroptosis, apoptosis, and/or necroptosis (78). A typical PANoptosome is composed of sensors (ZBP1, AIM2, RIPK1 and NLRP12), adaptors (ASC and FADD), and catalytic effectors (RIPK1, RIPK3, caspase-1 and caspase-8) (79, 80). The assembly of the PANoptosome is initiated by various factors, such as cellular stress or microbial infection. Once specific sensors are activated by these triggers, they initiate the assembly process of the PANoptosome. In this process, the interaction of conserved domains of the same or different types between proteins (such as CARD, DD, DED and PYD) provides the molecular scaffold for the assembly of the PANoptosome. The PANoptosome, once activated, initiates a cascade that activates downstream cell death effectors, culminating in a lytic form of inflammatory cell death (**Figure 2**) (81, 82).

Caspase-8 plays indispensable regulatory roles in PANoptosis and constitutes a fundamental part of the PANoptosome. As mentioned, caspase-8 facilitates apoptosis via the DR pathway, mitigates necroptosis by suppressing RIPK1, and further triggers pyroptosis by activating ASC and GSDMs. The dynamic activity of caspase-8 potentially shapes the plasticity of the intricate PANoptosis process. By precisely targeting the activity of caspase-8, scientists may develop innovative therapeutic approaches aimed at combating HCC.

## 4 Caspase-8 and inflammation

In addition to its regulatory function in PCD, caspase-8 also plays crucial roles in modulating inflammation, encompassing both anti-inflammatory and proinflammatory dual functions.

The anti-inflammatory role of caspase-8 is achieved primarily through promoting apoptosis, inhibiting necroptosis, and suppressing the inflammasome. Caspase-8 plays a crucial role as an important activator of apoptosis, and its activity is essential for apoptosis through the DR pathway. Apoptosis is a form of low-immunogenic PCD that eliminates damaged or infected cells, thus preventing excessive inflammation (83). Necroptosis is initiated when caspase-8 is incapacitated, leading to the activation of RIPK3 and MLKL triggered by TNFR activation. This leads to increased membrane permeability, liberating the cell contents, which triggers inflammatory responses. Caspase-8 can suppress necroptosis by cleaving RIPK1, thereby reducing inflammatory responses (84). Caspase-8 normally functions to inhibit the inflammasome. In dendritic cells (DCs), caspase-8 inhibition enhances the activation of the lipopolysaccharide-induced NLRP inflammasome and the production of IL-1β (85). This effect is related to MLKL but is distinct from the process of necroptosis. Another study revealed that the sole activation of MLKL can mediate NLRP3-dependent processes and the release of IL-1β without the involvement of GSDMD (86). In FADD^-/-^RIPK3^-/-^ myeloid cells, the conformation of inactive caspase-8 changes, potentially promoting the activation of caspase-1/11 and the release of IL-1β and IL-18 through autophagy and cathepsin-B pathway (87).

The proinflammatory effect of Caspase-8 is mediated through promoting pyroptosis and enhancing the maturation of IL-1β. Caspase-8 effectively promotes pyroptosis via both enzymatic and nonenzymatic pathways. Specifically, through its enzymatic activity, caspase-8 directly cleaves GSDMC, GSDMD, and GSDME. Alternatively, it may target downstream caspase-3, subsequently promoting the cleavage of GSDME, ultimately altering membrane permeability and causing the efflux of cellular contents. Furthermore, inactive caspase-8 functions as a protein scaffold, promoting the aggregation and activation of ASC and subsequently activating caspase-1 (88). Caspase-8 can activate caspase-1 via the inflammasome pathway, cleave pro-IL-1β and pro-IL-18, and release them through GSDMD-N pores (89). Caspase-8 can also promote the maturation of IL-1β independently of caspase-1. For example, in DCs, dectin-1 recognizes fungal and mycobacterial PAMPs, resulting in the formation of the mucosa-associated lymphoid tissue lymphoma translocation protein 1 (MALT1)-caspase-8-ASC inflammasome (90). This complex can cleave pro-IL-1β, and this conversion process remains unaffected by caspase-1 inhibitors.

Caspase-8 can also regulate inflammatory responses by promoting the nuclear factor-kappa B (NF-κB) pathway. NF-κB is a highly conserved transcription factor that participates in the regulation of gene expression, cytokine production, and cell survival, among other processes (91). In normal cells, NF-κB resides in the cytoplasm in an inactive state. The inhibitor of κB (IκB) protein binds to NF-κB, masking its nuclear localization site (92). NF-κB can be activated by various upstream signals such as TNF-α. For example, when TNF-α binds to TNFR1, the IKK complex is activated through adaptor proteins such as TRAF2, mediating the phosphorylation and ubiquitination of the inhibitory protein IκBα (93, 94). This process leads to the inhibition of IκB, which in turn enables the nuclear translocation of NF-κB, subsequently leading to the transcription of downstream genes. Fianco et al. reported that caspase-8 was highly expressed in glioblastoma, which promoted the activation of NF-κB, further increasing the expression of IL-1β, IL-6, and IL-8 (24). In adipocytes, the inhibition of caspase-8 can lead to the downregulation of NF-κB signaling, subsequently causing a decrease in TNF-α levels (83). Davidovich et al. reported that the caspase-8-FADD-RIPK1 complex enhances the production of IL-6 via the NF-κB pathway (58). Caspase-8 functions as a scaffold to promote the aggregation of RIPK1 and FADD, whereas cFLIP inhibits this process owing to its lower affinity for FADD. However, Xia et al. reported that in prostate cancer, caspase-8 upregulates the NF-κB pathway to promote the upregulation of downstream IL-8. This process is independent of its proteolytic activity but requires interaction with cFLIP (95). Existing studies suggest that the assembly of caspase-8 scaffolding is an indispensable initial step for the formation of DISC and NF-κB activation. However, caspase-8’s catalytic activity seems redundant for NF-κB activation and the induction of subsequent cytokines (96). How caspase-8 activates RIPK1 and phosphorylates IκBα remains to be clarified.

## 5 Caspase-8 and HCC

Given the intricate functions of Caspase-8, it plays a pivotal role in the pathogenesis of HCC. As previously discussed, CLDs stemming from diverse causes have been established as significant high-risk factors for HCC (3). Moreover, the chronic hepatocyte apoptosis induced by CLDs and their subsequent regenerative process serve as pivotal mechanisms underlying the pathogenesis of HCC. Caspase-8 can facilitate the timely clearance of impaired hepatocytes, thereby preserving liver health. However, an extended period of hepatocyte apoptosis can be detrimental. Boege et al. reported that caspase-8-induced hepatocyte proliferative stress is a risk factor for HCC independent of the aetiology of CLDs and that the caspase-8-dependent DNA damage response (DDR) relies on the nonapoptotic function of caspase-8 rather than its catalytic activity (8). The caspase-8-FADD-cFLIP-RIPK1 complex coordinates the regulation of cell fate, inflammasome activation, NF-κB activation, cytokine production, and other processes. Additionally, full-length caspase-8, rather than its catalytic activity, can promote the phosphorylation of H2AX (γH2AX) (8). γH2AX is a marker of DNA damage that can facilitate the initiation of DNA damage repair mechanisms (97). Therefore, caspase-8 deficiency or silencing can confer antiapoptotic ability to cells and promote the accumulation of DNA replication errors and mutations, thereby advancing the progression towards HCC.

The nonapoptotic functions of caspase-8 also play a significant role in the progression of HCC. Research has indicated that caspase-8 is overexpressed in certain malignancies, such as HCC, indicating that these tumors can resist apoptosis when caspase-8 is highly expressed (98). Consistently, cFLIP is frequently constitutively overexpressed in HCC cell lines, and its overexpression is associated with an unfavorable tumor prognosis (99). cFLIP is modulated by multiple signaling pathways such as the NF-κB pathway (100). In HCC with high expression of caspase-8, cFLIP not only blocks caspase-8-mediated apoptosis but also modulates NF-κB pathways through the caspase-8-FADD-cFLIP-RIPK1 complex, promoting the survival and drug resistance of HCC (101, 102).

Overall, caspase-8 plays a significant role in the progression of HCC, and additional research is warranted to elucidate the precise mechanisms through which caspase-8 influences HCC development.

## 6 Caspase-8 and the HCC TME

The TME constitutes an intricate web of diverse cellular and noncellular elements that are intertwined through sophisticated interactions (103). This intricate network profoundly influences tumor initiation, progression, invasive behavior, and resistance to therapeutic interventions. The immunosuppressive TME (ITME) is a critical component of the TME that functions to suppress immune functions. Compared with diverse cellular and acellular elements, the ITME promotes tumor growth, invasion, and metastasis through intricate interactions while simultaneously inhibiting the body’s antitumor immune response (104, 105). This intricate milieu holds paramount importance in orchestrating the pathogenesis, progression, metastasis, and development of drug resistance in HCC, underscoring its pivotal role in shaping the tumor’s biological behavior and therapeutic responsiveness of tumors. Caspase-8, which serves as a central hub in multiple signaling pathways, plays a pivotal role in the HCC TME, regulating HCC tumor immunity.

### 6.1 Caspase-8 and TAM

Tumor-associated macrophages (TAMs), which predominantly originate from circulating monocytes, are instrumental in the progression of HCC, inflammatory response regulation, and immune suppression (106, 107). TAMs can polarize into the M1/M2 phenotype. Furthermore, owing to the plasticity and heterogeneity of macrophages, M1 and M2 TAMs can interconvert on the basis of the specific conditions within the TME (108, 109). Classically activated M1 TAMs are primarily induced by factors such as interferon gamma (IFN-γ), IL-12, and lipopolysaccharide (LPS) (110). They secrete proinflammatory cytokines, thereby stimulating immune surveillance functions. In contrast, M2 TAMs are induced by factors such as IL-4, IL-10, and TGF-β (111). M2 TAMs release inhibitory cytokines and chemokines, thereby facilitating adverse biological processes such as tumor proliferation, tumor angiogenesis, and immune evasion. In the HCC TME, the majority of cytokines tend to promote the polarization of TAMs towards the M2 phenotype, especially in advanced stages, thereby facilitating tumor progression (112). RNA-seq data revealed that HCC samples with higher levels of M2 TAMs had poorer prognoses, and several HCC prognostic markers specific to M2 TAMs were identified (113). Specifically, M2 TAMs actively promote the generation and construction of neovascular networks within tumors by secreting vascular endothelial growth factor (VEGF), providing nutritional support for HCC cell growth (114). Additionally, they release metalloproteinases (MMPs) to degrade the extracellular matrix, thereby enhancing the invasive capabilities of HCC cells (115). Moreover, M2 TAMs further consolidate the immune evasion mechanisms of HCC by suppressing the activity of antitumor immune cells such as natural killer (NK) cells and cytotoxic T lymphocytes (CTLs) and secreting inhibitory cytokines such as TGF-β and IL-10 (116). Notably, TGF-β not only participates in immune suppression but also promotes epithelial−mesenchymal transition in HCC cells. This process augments the migratory and invasive capabilities of HCC cells and may confer cancer stem cell-like properties to them (117).

Caspase-8 is instrumental in guiding macrophage differentiation. In bone marrow cell lines, caspase-8 facilitates transient activation of the NF-κB pathway through its scaffolding function, thereby promoting M0 differentiation. A deficiency in caspase-8 can block M0 differentiation (118). Cuda et al. revealed that caspase-8-mediated regulation of macrophages governs TLR activation and M1 polarization through a RIPK1-dependent mechanism. Inhibition of caspase-8 can lead to the activation of RIPK1/RIPK3, resulting in increased expression of the costimulatory factor CD86 and increased production of IL-1β upon TLR activation (119). Another study reported that CCL2 and IL-6 in the TME can promote the expression of full-length caspase-8 by inducing cFLIP but inhibit the apoptotic function of caspase-8. Subsequently, caspase-8 may function as a scaffold to promote M2 polarization, although the specific mechanism remains unclear (120). Furthermore, Caspase-8 can lead to a decrease in Kupffer cells (hepatic macrophages, KCs) after partial hepatectomy for HCC, which will facilitate tumor cell proliferation and increase the risk of HCC recurrence (121). Mechanistically, caspase-8 is activated through the TNF-α pathway, which promotes the assembly of complex IIb, thereby facilitating KC apoptosis. Additionally, it can also induce KC pyroptosis through RIPK3-dependent caspase-1 activation. Notably, this process does not involve necrosis, and there is no increase in MLKL phosphorylation. A reduction in KCs promotes the recruitment of circulating monocytes, which differentiate into Ly6C^low^ macrophages, facilitating the resolution of inflammation, suppressing T-cell activity, and promoting angiogenesis (121, 122).

### 6.2 Caspase-8 and T cells

T cells, which stem from lymphoid progenitor cells, undergo multiple developmental stages in the thymus, ultimately differentiating into CD4^+^/CD8^+^ single-positive T cells. CD8^+^ T cells, also called cytotoxic T lymphocytes (CTLs), exhibit moderate affinity for the class I major histocompatibility complex (MHC I). Upon recognition of MHC I antigen stimulation by the T-cell receptor (TCR), CD8^+^ T cells become activated and proliferate, killing target cells through various mechanisms (123). These include the secretion of cytokines such as IFN-γ and TNF-α, the release of perforin and granzymes, and the induction of apoptosis via Fas/FasL interactions (124, 125). In the HCC TME, CD8^+^ cells perform immune surveillance functions, but their frequency is often lower than that in nontumorous regions. The exhaustion of CD8^+^ T cells has been linked to a decrease in overall survival rates in patients with HCC (126–129). CD4^+^ T cells are also called T helper (Th) cells. After recognizing MHC II antigens, naive CD4^+^ T cells (Th0) can specialize by differentiating into various subsets of CD4^+^ T cells. Based on their differential expression of transcription factors, CD4^+^ T cells can be classified into various subsets (130). These diverse CD4^+^ T-cell subsets are capable of secreting both proinflammatory and anti-inflammatory cytokines. Regulatory T cells (Tregs, CD4^+^CD25^+^Foxp3^+^ T cells) are essential cells that contribute to immune suppression within the TME (131). Tregs exert immunosuppressive effects primarily through various mechanisms, including the secretion of inhibitory cytokines (such as TGF-β and IL-10) and the expression of inhibitory cell-surface molecules and competitive inhibitory cytokines (such as CTLA-4 and PD-1) (132–134). Suthen et al. reported that exhausted CD8^+^ T cells and Treg cells are enriched in the hypoxic HCC TME, whereas active CD8^+^ T cells are excluded (135).

Caspase-8 performs a vital function in regulating T-cell homeostasis and mediating T-cell immune responses. Salmena et al. reported that mutations in caspase-8 can lead to a decrease in the frequency of peripheral T cells, despite normal thymic cellular development (136). Furthermore, these mutations result in the absence of the ability of T cells to produce IL-2 and respond to exogenous IL-2. Similarly, in mice, the specific deletion of caspase-8 in T cells can lead to an age-dependent and fatal immune dysregulation (137). Caspase-8 also has differential effects on Tregs under diverse conditions. Under homeostatic conditions, the caspase-8-mediated DR pathway restricts the population of effector T cells (CCR7^low^, PD-1^high^, CTLA-4^low^, ICOS^high^, TIGIT^high^). When caspase-8 expression is specifically inhibited in Treg cells, the function of Tregs remains normal, and the number of effector Tregs increases (138). During inflammation, caspase-8 promotes the survival of Tregs, whereas the inhibition of caspase-8 leads to Treg death and excessive immune activation. Conventional T cells and Tregs exhibit different sensitivities to necroptosis (139). Compared with conventional T cells, Tregs are more sensitive to emricasan, a caspase-8/cFLIP heterodimer inhibitor, which results in high expression of RIPK3 and MLKL, thereby inducing necroptosis (138). Similarly, Carlos et al. reported that Tregs are also more sensitive to apoptosis than conventional T cells. The level of cFLIP in Tregs is significantly lower than that in control cells, and this deficiency in cFLIP markedly increases the levels of active caspase-3 and caspase-7 in Tregs, thereby increasing the rate of Treg apoptosis (140). Stimulation with TGF-β can increase the expression of cFLIP in Tregs. Caspase-8-related PCD can also regulate T-cell responses. For example, RNA-seq data analysis has revealed that the expression of the key necroptosis factors RIPK1, RIPK3, and MLKL is significantly correlated with the infiltration of HCC CD8^+^ T cells (141). Furthermore, poly (ADP−ribose) polymerase inhibitor (PARPi) treatment can promote pyroptosis via the GSDMC−caspase-8 pathway in triple-negative breast cancer cells and mediate the infiltration of CD8^+^ T cells in the TME (142). However, IL-18 produced by pyroptosis has also been linked to poor outcomes in HCC patients, as HCC patients with positive IL-18 receptor expression exhibit lower survival rates (143). Li et al. reported that TLR2 can inhibit caspase-8-mediated IL-18 production and increase the number of functional CD8^+^ T cells, thereby inhibiting HCC (144). In conclusion, the complex interplay between caspase-8 and T cells needs definitive and direct evidence to elucidate the precise mechanisms by which caspase-8 regulates T cells, especially Tregs, within the HCC TME.

### 6.3 Caspase-8 and NK cells

NK cells are integral to the innate immune system and possess potent cytotoxic capabilities. NK cells can directly kill target cells by recognizing specific surface features of these cells without relying on antigen recognition or MHC restriction (such as tumor cells with downregulated MHC I) (145, 146). In addition, NK cells are adept at secreting a diverse array of cytokines, such as IFN-γ, GM-CSF, and the chemokines CCL4 and CCL5, which play intricate roles in coordinating immune responses, enhancing inflammatory reactions, and recruiting other immune cells (147). However, within the TME, the activity of NK cells is often severely suppressed (148). Many adverse factors within the TME, including hypoxia, adenosine, TGF-β, and prostaglandin E2, can effectively diminish the activity and function of NK cells (149). Adding to this complexity, the presence of immunosuppressive cell populations such as TAMs and Tregs further exacerbates the inhibition of NK cell immune function (150). Immunotherapy based on NK cells represents a highly promising direction in the field of HCC treatment (151, 152). Xiao et al. reported that Siglec-9 and its ligands are highly expressed on NK cells, inhibiting their antitumor immunity and correlating with poor outcomes of patients with HCC (153). They reported that the small molecule inhibitor MTX-3937, which is designed to target Siglec-9, significantly improves NK cell function and enhances HCC immune surveillance.

Caspase-8 is one of the crucial factors through which NK cells perform their cytotoxic functions. Studies have reported that NK cells can trigger apoptosis in target cells by liberating perforin and granzymes and by activating DRs such as CD95/FAS and TNF-related apoptosis-inducing ligand (TRAIL) receptors on the surface of target cells, thereby activating caspase-8-mediated apoptosis (154–156). Prager et al. reported that NK cells primarily mediate cell death through granzyme B during the first kill. In contrast, in subsequent killing processes, they shift to DR-mediated apoptosis. Prolonged cell-to-cell contact precipitates a reduction in granzyme B and perforin within NK cells, coupled with an increase in CD95L on their surface. This shift triggers a transition in the killing pathways (157). Zhao et al. reported that pyroptosis is the predominant mode of hepatocyte death in patients with HBV-related acute-to-chronic liver failure. In hepatocytes with HBV reactivation, the absence of MHC-I molecules activates cytotoxic NK cells, subsequently triggering GSDMD/caspase-8-dependent pyroptosis in hepatocytes (158). In addition, caspase-8 can modulate the immune response by curbing the overproliferation of NK cells and CD8^+^ T cells during the expansion phase. Caspase-8^-/-^RIPK3^-/-^ and caspase-8^-/-^RIPK^1-/-^RIPK3^-/-^ mice exhibit higher levels of mouse pathogen murine cytomegalovirus-specific NK cells and CD8^+^ T cells (159).

### 6.4 Caspase-8 and dendritic cells

As the body’s most powerful antigen-presenting cells, DCs can be classified on the basis of their origin into myeloid DCs (mDCs), plasmacytoid DCs (pDCs), or monocyte-derived DCs (MoDCs) (160, 161). DCs exhibit high levels of MHC I and MHC II, as well as the costimulatory molecules CD80 and CD86 and the adhesion molecules CD40 and CD54 on their surfaces (162, 163). This enables DCs to efficiently capture, process, and present antigens after recognizing them. In the TME, DCs recognize, process, and present tumor antigens, activating T-cell-mediated antitumor immune responses, making them one of the important targets for tumor immunotherapy (164). DC dysfunction is a pivotal contributing factor to the formation of the ITME. Galarreta et al. reported that the activation of β-catenin in HCC can elicit immune evasion and decrease the effectiveness of anti-PD-1 therapy (161). Mechanistically, the activation of b-catenin leads to a reduction in the recruitment of CD103^+^ DCs. This decrease in DC numbers subsequently results in a decrease in HCC-specific CD8^+^ T cells. The overexpression of the chemokine CCL5 can reverse this trend, reinstating immune surveillance. Furthermore, in the HCC TME, hypoxic conditions increase the expression of hypoxia-inducible factor-1α (HIF-1α), leading to the overexpression of CD47 and the inhibition of CD103^+^ DC function (165). By blocking CD47, the capacity of CD103^+^ DCs to take up tumor DNA is increased, thereby promoting the secretion of CXCL9 and IL-12, activating the cyclic GMP-AMP synthase (cGAS)-stimulator of interferon genes (STING) pathway, and facilitating the recruitment and activation of NK cells within HCC (166). Single-cell RNA sequencing has revealed that in the Scirrhous HCC (SHCC) TME, hypoxic conditions trigger the upregulation of secreted phosphoprotein 1 (SPP1), which inhibits DC function and impedes T-cell activation through the SPP1−CD44 axis (167).

In DCs, caspase-8 can initiate the maturation of IL-1β. The administration of doxorubicin can trigger the release of IL-1β, a process intimately linked to caspase-8, which can be inhibited by caspase-8 inhibitors (168). Caspase-8-mediated maturation and release of IL-1β rely on the Toll/IL-1R domain-containing adapter-inducing IFN-γ (TRIF). TRIF is crucial in TLR4 signaling and potentially engages in the assembly of caspase-8 signaling complexes. Furthermore, in addition to antigen presentation, immature DCs also exhibit the ability to induce cell death, which is not possible for mature DCs. Vanderheyde et al. reported that MoDCs exhibit a caspase-8-dependent and FADD-independent tumor killing activity (169). This type of apoptosis does not involve the DR pathway, and blocking TNF/TNFR, CD95/CD95 ligand, or TRAIL/TRAIL receptor interactions cannot reverse this process. Conversely, overexpression of Bcl-2 increases the resistance of tumor cells. Varga et al. reported that MoDCs induce caspase-8-dependent apoptosis in Jurkat cells, and that this process can be completely blocked by caspase-8 inhibition (170).

## 7 Targeting caspase-8 in HCC therapy

Owing to its crucial role in PCD and tumor immunity, targeting caspase-8 presents new opportunities for treating HCC. Chemotherapy and radiotherapy precisely target HCC cells by causing DNA damage, which then initiates cell death. Apoptosis is the predominant pathway for this form of cell death. In HCC, the enzymatic activity of caspase-8 may be suppressed, which allows cancer cells to undergo apoptosis. Consequently, activating caspase-8 can induce apoptosis in these cells, thereby inhibiting the progression and metastasis of HCC. Adiponectin improves HCC partially by increasing the activity of p53 and the expression of TRAIL, and by increasing the levels of caspase-8 and caspase-3, thus promoting the apoptosis in of HCC cells (171). Che et al. reported that Cullin-associated NEDD8-dissociated 1 (CAND1) is highly expressed in HCC and can serve as an independent prognostic factor for HCC patients (172). CAND1 regulates the activity of caspase-8, and knocking down CAND1 can activate caspase-8 and amplify the apoptotic signal through the mutual activation of caspase-8-receptor interacting protein 1 (RIP1), promoting HCC apoptosis. Im et al. reported that HCC highly expresses DNA damage-induced apoptosis suppressor (DDIAS), which inhibits TRAIL-induced apoptosis by suppressing caspase-8 (173). Mechanistically, DDIAS binds to the DED of FADD, inhibiting the recruitment and oligomerization of caspase-8. Furthermore, DDIAS can promote the activation of P90 ribosomal S6 kinase 2 (RSK), leading to the phosphorylation of caspase-8 at the S227 site and promoting the ubiquitination of caspase-8. DDIAS knockdown enhances the sensitivity of HCC to the TRAIL-caspase-8 apoptosis pathway. Jin et al. reported that the long non-coding RNA (LncRNA) CASC2 promotes the expression of caspase-3/8 by acting as a sponge for miR-24 and miR-221, thereby influencing TRAIL-induced tumor cell apoptosis and drug resistance and ultimately improving TRAIL resistance in HCC (174). El-Demiry et al. reported that combined treatment with cisplatin and sunitinib significantly increased the levels of caspase-9 and caspase-8 while significantly reducing RIPK3 levels. Despite reducing necroptosis, sunitinib has been shown to intensify cisplatin-induced apoptosis and amplify oxidative stress, thereby resulting in increased cytotoxicity against HepG2 cells (175). As a key modulator of caspase-8 enzymatic activity, cFLIP is overexpressed in certain HCC patients, contributing to resistance to apoptosis. Inhibition of cFLIP is one of the proposed means to increase the responsiveness of HCC to chemotherapeutic drugs. Luan et al. reported that rocaglamide enhances the sensitivity of HepG2 cells to TRAIL by reducing the expression levels of cFLIP (176). Carlisi et al. reported that suberoylanilide hydroxamic acid (SAHA) can promote the expression of DR5 and inhibit cFLIP, facilitating the rapid activation of caspase-8 induced by TRAIL and HepG2 cell apoptosis, with no effect on primary human hepatocytes (177). Jeon et al. reported that the combined therapy utilizing maritoclax and TRAIL significantly induced apoptosis in HCC cells. Mechanistically, maritoclax enhances the susceptibility of HCC to TRAIL-mediated apoptosis through the downregulation of cFLIP by miR-708 (178). Decoy receptor 3 (DcR3) is overexpressed in various malignant tumors (179). Liang et al. reported that knocking down DcR3 can inhibit the transcription of cFLIP, promote the expression of caspase-8, and induce apoptosis in HepG2 cells (180). Furthermore, the short hairpin DcR3 can also inhibit the activation of the IKK-mediated NF-κB pathway. Overall, reactivating apoptosis in HCC cells by activating caspase-8 or inhibiting its negative regulatory factors could be an effective treatment option for HCC.

In addition to cFLIP, another key factor affecting caspase-8 targeted therapy is caspase-10. Caspase-10 also has two DED domains and is the only caspase similar to caspase-8 (181). Furthermore, caspase-10 possesses the same enzymatic active center QACQG as caspase-8 (182). These structural similarities make it challenging to target caspase-8 selectively. Z-IETD-FMK is an effective caspase-8 specific inhibitor with the sequence Z-Ile-Glu-Thr-Asp-FMK, which highly matches the substrate recognition site of caspase-8, thus allowing it to bind specifically to caspase-8 and inhibit its activity (183). Zhang et al. reported that overexpression of Z-IETD-FMK can inhibit caspase-8 and reduce apoptosis in HCC cells (184). However, there have been no reports of effective caspase-8 specific activators to date, which implies that caspase-8 activators might also inadvertently activate caspase-10. The upregulation of caspase-10 can also modulate the extrinsic apoptotic pathway. Qi et al. reported that bufalin and cinobufagin can promote apoptosis in HCC cells, and both Z-IETD-FMK and Z-AEVD-FMK (caspase-10 inhibitors) can suppress this process. This will affect the accurate assessment of the effects on caspase-8 (185). In addition, the activation of caspase-10 has a regulatory effect on the activity of caspase-8. Mohr et al. reported that 5-fluorouracil can induce caspase-8-mediated apoptosis in tumor cells (186). In this process, caspase-10 is upregulated in an ataxia telangiectasia mutated and Rad3-related kinase (ATR)-dependent manner and forms a complex known as the FADDosome with caspase-8, FADD, RIPK1, and TRAF2. This complex mediates the ubiquitination and degradation of cFLIP by TRAF2, leading to the activation of caspase-8. Conversely, in tumor cells lacking caspase-10, TRAF2, or ATR, the mode of cell death shifts to a more effective autocrine/paracrine mode, initiated by another complex, the FLIPosome, which results in the processing of cFLIPL and the production of TNF-α, promoting p53-independent apoptosis (186). Zhang et al. reported that the POK erythroid myeloid ontogenic factor (Pokemon) is overexpressed in HCC cells (187). In HepG2 cells with silenced Pokemon, treatment with oxaliplatin can activate caspase-10 and caspase-8, promoting the release of the active fragments p18 and p10 of caspase-8. In contrast, in another study, Horn et al. reported that in HeLa cells, caspase-10 negatively regulates DISC-mediated caspase-8 activation and promotes the activation of the NF-κB pathway, converting the cell’s response to CD95 into cell survival (96). Caspase-8 recruits caspase-10 through a scaffolding function, and the activation of NF-κB also depends on the scaffolding functions of both caspase-8 and caspase-10. Inhibition of caspase-10 reduces the expression of cytokines and facilitates apoptosis in tumor cells. Considering the similarities and interactions between caspase-10 and caspase-8, the development of new compounds or biomolecules that can specifically activate or inhibit caspase-8 without affecting caspase-10 may become a key direction for future research. This line of research is expected to provide a solid scientific foundation for the development of more effective treatment strategies for HCC. Furthermore, it should be noted that the absence of caspase-10 in rodents means that caution is warranted when extrapolating results related to caspase-8 from mouse studies to human contexts (188, 189).

In addition to apoptosis, the modulation of necroptosis and pyroptosis pathways by targeting caspase-8 holds immense potential as a research direction for enhancing the sensitivity of HCC cells to treatments. Xiang et al. reported that high expression of Connexin32 (Cx32) enhances the antiapoptotic capability of HCC cells, promoting the malignant progression of HCC (190). In HCC, which is characterized by high Cx32 expression, inhibiting caspase-8 to induce necroptosis represents a promising therapeutic strategy. Cx32 can bind to Src and promote Src-driven phosphorylation and inhibition of caspase-8. It can also inhibit caspase-8 activity by increasing c-FLIP expression and reducing FADD expression. The overexpression of Cx32 significantly enhances the therapeutic effect of shikonin, an activator of necroptosis (191). However, necroptosis in HCC is a double-edged sword; although necroptosis can kill HCC cells, the DAMPs and cellular debris produced by necroptosis may exacerbate the inflammatory response within the HCC TME, promoting angiogenesis and tumor metastasis (192, 193). Vucur et al. reported that the NF-κB signaling is a major cause of promoting hepatocyte necroptotic inflammation and HCC (194). In hepatocytes with naturally low expression of RIPK3 and tumors with low RIPK3 expression, when caspase-8 is underexpressed, MLKL phosphorylation is incomplete, and cells do not die immediately but instead maintain membrane leakage and inflammation for a long time, promoting the occurrence of tumors. This prolonged subnecrotic apoptosis is closely related to the simultaneous activation of NF-κB. Necroptosis without NF-κB activation does not promote the occurrence of liver cancer in mice. Therefore, targeting the reprogramming of necroptosis during caspase-8 inhibition may be a strategy for treating RIPK3-low-expressing HCC. Furthermore, determining how to specifically modulate caspase-8 inhibition and necroptosis in HCC cells to minimize the impact on normal cells is essential. Necroptosis can cause chronic inflammation in hepatocytes and contribute to liver fibrosis (195). Abnormal activation of RIPK3 due to caspase-8 deficiency can lead to midgestational death in mouse embryos, whereas caspase-8^-/-^RIPK3^-/-^ mice can survive but accumulate abnormal T cells (196). Targeting RNA changes that are specific to HCC may prove to be an effective approach. Visalli et al. reported a triple-miRNA signature (miR-371-5p, miR-373, and miR-543) that is overexpressed in HCC tissues and promotes the development of HCC (197). These three miRNAs can directly bind to the 3’UTR of *CASP8*, specifically inhibiting the expression of caspase-8 and promoting necroptosis in HCC cells. Targeting caspase-8-associated pyroptosis is also a potential direction for HCC treatment. Several pyroptosis-associated gene models that include *CASP8* have been established for predicting the outcome of patients with HCC (198–200). Cui et al. reported that reuterin can increase the sensitivity of HCC to sorafenib. Mechanistically, reuterin promotes pyroptosis via the cGAS−STING pathway and upregulates caspase-8. Activation of the STING pathway promotes the necroptosis and pyroptosis pathways. However, the upregulation of caspase-8 inhibits necroptosis and further promotes HCC pyroptosis through the caspase-8-GSDMD pathway (201). This interplay between necroptosis and pyroptosis, modulated by caspase-8, underscores the complexity of cellular death mechanisms in HCC and highlights the potential for novel therapeutic interventions that could selectively target these pathways.

In addition to inducing PCD, caspase-8 also regulates the TME through multiple mechanisms. DAMPs and proinflammatory cytokines released during PCD, such as necroptosis and pyroptosis can facilitate the infiltration of immune cells, potentially converting “cold” tumors into “hot” tumors and increasing the effectiveness of immunotherapy (202). However, the released DAMPs and cellular debris may trigger or exacerbate inflammatory responses within the HCC TME (193, 203). This inflammatory response may promote HCC cell proliferation, invasion, and metastasis, creating favorable conditions for HCC development and progression. Additionally, caspase-8 activates the NF-κB pathway through its scaffolding function, promoting the expression of various cytokines. Fianco et al. reported that caspase-8 activates the NF-κB pathway, promoting the expression of various cytokines, such as IL-1, IL-6, and VEGF (24). This activation fosters an inflammatory TME and neovascularization in glioblastoma, enhancing its resistance to temozolomide. In another study, Tsai et al. reported that magnolol promoted the enzymatic activity of caspase-8 and the activation of the apoptotic pathway while simultaneously inhibiting the NF-κB pathway, thus reducing the expression of VEGF and MMP-9 in HCC (204).

Furthermore, caspase-8 plays a regulatory role in the differentiation, homeostasis, and function of various immune cells. A multidimensional study revealed that high expression of caspase-8 is associated with poor outcomes in HCC patients and that patients with high caspase-8 expression have a relatively high mutation frequency of p53. In addition, caspase-8 activity is influenced by various immune cells in the HCC TME, such as CD4^+^ T cells, CD8^+^ T cells, M2 macrophages, and NK cells (205). Some factors not only affect the activity of caspase-8 but also regulate immune cells. For example, the knockdown of DcR3 not only reduces the transcription of cFLIP and caspase-8 but also promotes the differentiation of Th0 cells into Th1 cells, inhibits the differentiation of Th2 and Treg cells, and enhances tumor immunity in HCC (180, 206). Nevertheless, the specific research progress on the regulation of caspase-8 by immune cells in the HCC TME is insufficient to elucidate the detailed mechanisms involved, indicating that numerous areas require further in-depth investigation (**Table 1**).

**Table 1:** Modulators Targeting Caspase-8 in Hepatocellular Carcinoma.

Modulators	Caspase-8	HCC	Mechanisms	References
Adiponectin	Increase	Inhibit	Enhancing caspase-8 and caspase-3 levels to promote apoptosis in HCC cells.	(171)
CAND1		Inhibit	Activating caspase-8-mediated apoptosis in HCC cells.	(172)
LncRNA CASC2		Inhibit	Inhibiting miR-24 and miR-221 to promote caspase-8 and TRAIL-induced apoptosis in HCC.	(174)
Sunitinib		Inhibit	Increasing caspase-8 and caspase-9 to promote HCC apoptosis and inhibit necroptosis.	(175)
SAHA		Inhibit	Promoting DR5 and inhibiting cFLIP to enhance apoptosis in HepG2 cells	(177)
Maritoclax		Inhibit	Inhibiting cFLIP through miR-708 and promoting apoptosis.	(178)
5-fluorouracil		Inhibit	Promoting FADDosome and mediating cFLIP ubiquitination.	(185)
Reuterin		Inhibit	Up-regulating caspase-8 to inhibit necroptosis and promoting the caspase-8-GSDMD pathway to facilitate pyroptosis in HCC.	(201)
Magnolol		Inhibit	Promoting caspase-8-mediated pyroptosis and inhibiting the NF-κB pathway to reduce the expression of VEGF and MMP-9.	(203)
DDIAS	Reduce	Promote	Inhibiting caspase-8 recruitment and promoting caspase-8 ubiquitination.	(173)
Rocaglamide		Promote	Inhibiting cFLIP to promote TRAIL-induced apoptosis.	(176)
DcR3		Promote	Promoting cFLIP transcription and NF-κB pathway.	(180)
Z-IETD-FMK		Promote	Inhibiting caspase-8 expression to reduce apoptosis in HCC cells.	(184)
Cx32		Inhibit	Promoting cFLIP and reducing FADD to inhibit caspase-8.	(191)
miR-371-5p, miR-373, miR-543		Promote	Binding to the 3'UTR of *CASP8* gene to inhibit the expression of caspase-8.	(197)

CAND1, cullin-associated NEDD8-dissociated 1; Caspase-8/9, cysteinyl aspartate specific proteinase 8/9; cFLIP, cellular-FLICE inhibitory protein; Cx32, connexin32; DcR3, decoy receptor 3; DDIAS, DNA damage-induced apoptosis suppressor; DR, death receptor; FADD, Fas-associated death domain; GSDMD, gasdermin; HCC, hepatocellular carcinoma; LncRNA, long non-coding RNA; MMP-9, metalloproteinase- 9; NF-κB, nuclear factor-kappa B; SAHA, suberoylanilide hydroxamic acid; TRAIL, TNF-related apoptosis-inducing ligand; VEGF, vascular endothelial growth factor.

## 8 Conclusion

HCC remains as a formidable global health issue because of its elevated mortality rates and the paucity of effective therapeutic options. A thorough understanding of the complex molecular mechanisms that underpin the development of HCC is imperative for identifying innovative therapeutic avenues. Among the myriad of factors implicated in HCC pathogenesis, caspase-8 has emerged as a versatile protein with pivotal roles in modulating PCD, inflammation, and the TME.

Caspase-8 is a pivotal factor in DR pathway apoptosis, yet its dysregulation in HCC frequently culminates in chemo- and radio resistance. In addition to fostering apoptosis, caspase-8 exerts a regulatory influence on necrosis and pyroptosis, significantly contributing to the intricate PANoptosis process. In HCC, the activation of caspase-8-mediated PCD can increase the efficacy of conventional therapeutic strategies. Conversely, the emanation of DAMPs and inflammatory mediators from PCD may instigate inflammatory cascades within the TME, potentially facilitating HCC invasion and metastasis.

The TME serves as a crucial determinant of the progression and treatment response of HCC. Caspase-8 regulates the differentiation, recruitment, homeostasis, and functionality of various immune cells within the HCC TME, underscoring its potential as a therapeutic target. However, current research fails to provide a comprehensive understanding of the intricate mechanisms that drive these processes. Additional studies are imperative to shed light on the precise role that caspase-8 plays in the HCC TME.

Targeting caspase-8 in HCC therapy presents a promising yet challenging avenue. Strategies involving the reactivation of caspase-8 in apoptosis-resistant HCC cells, as well as the promotion of necroptosis and pyroptosis, are actively being explored. The development of small molecule inhibitors, antisense oligonucleotides, and other modalities aimed at modulating caspase-8 activity is a promising area of research. Additionally, the discovery of predictive biomarkers capable of predicting responses to caspase-8-targeted therapies could personalize treatment and improve patient outcomes. However, current research remains in its infancy. The regulatory role of caspase-8 within the complex TME of HCC is not yet fully understood, necessitating further in-depth investigation to elucidate the mechanisms by which targeting the enzymatic and scaffolding functions of caspase-8 can modulate HCC. Moreover, developing caspase-8 activators and inhibitors with high specificity and selectivity presents a significant challenge. Ensuring the effective delivery of these drugs to the tumor tissue and their ability to penetrate the richly vascularized TME of HCC is a technical hurdle that must be overcome.

In conclusion, the intricate involvement of caspase-8 in HCC pathophysiology positions it as a potential therapeutic target. Its regulatory roles in PCD, inflammation, tumor immunity, and the TME make it a compelling target for novel therapeutic strategies. Future research endeavors should focus on deciphering the exact mechanisms through which caspase-8 modulates the behavior of various immune cells within the HCC TME. It is essential to conduct clinical studies to assess the safety and efficacy of caspase-8-targeted therapies among HCC patients. With a deeper understanding of the functions and regulatory mechanisms of caspase-8, we can develop more effective treatments to improve the survival rates of HCC patients.

## 9 Methods

We conducted a systematic literature search using PubMed, EMBASE, Web of Science, and CENTRAL within the Cochrane Library without date or language limitations. The search terms used were “(Hepatocellular carcinoma OR HCC) AND (Caspase-8)”, “(Apoptosis) AND (Caspase-8)”, “(Necroptosis) AND (Caspase-8)”, “(Pyroptosis) AND (Caspase-8)”, “(PANoptosis) AND (Caspase-8)”, “(Hepatocellular carcinoma OR HCC) AND (Apoptosis)”, “(Hepatocellular carcinoma OR HCC) AND (Necroptosis)”, “(Hepatocellular carcinoma OR HCC) AND (Pyroptosis)”, “(Hepatocellular carcinoma OR HCC) AND (PANoptosis)”, “(Hepatocellular carcinoma OR HCC) AND (Tumor microenvironment OR TME)”, “(Caspase-8) AND (Tumor microenvironment OR TME)”, “(Caspase-8) AND (Caspase-10)”, “(Hepatocellular carcinoma OR HCC) AND (Caspase-10)”.
